# METTL16 predicts a favorable outcome and primes antitumor immunity in pancreatic ductal adenocarcinoma

**DOI:** 10.3389/fcell.2022.759020

**Published:** 2022-09-09

**Authors:** Liting Lu, Dandan Zheng, Junchi Qu, Yanyan Zhuang, Juanfei Peng, Sihua Lan, Shineng Zhang, Fengting Huang

**Affiliations:** ^1^ Department of Gastroenterology, Sun Yat-sen Memorial Hospital, Sun Yat-sen University, Guangzhou, China; ^2^ Guangdong Provincial Key Laboratory of Malignant Tumor Epigenetics and Gene Regulation, Sun Yat-Sen Memorial Hospital, Sun Yat-Sen University, Guangzhou, China

**Keywords:** pancreatic ductal adenocarcinoma, METTL16, prognostic biomarker, tumor microenvironment, N6-methyladenosine

## Abstract

Pancreatic carcinogenesis is a complicated and multi-step process. It is substantially assisted by N6-methyladenosine (m^6^A) RNA modification, especially when mutations of driver genes (KRAS, TP53, CDKN2A, and SMAD4) occur. However, the underlying mechanism remains obscure. In this research, we identified m^6^A regulators as potential biomarkers when mutations of driver genes occur, and investigated the role of these m^6^A candidates in pancreatic ductal adenocarcinoma (PDA). We first estimated the abnormal expression patterns of potential m^6^A regulators when all the driver genes are mutated, using The Cancer Genome Atlas and Gene Expression Omnibus databases. METTL16, an m^6^A“writer,” was chosen as a unique candidate of PDA, owing to its markedly differential expression under mutations of all driver genes (KRAS, TP53, CDKN2A, and SMAD4) and its favorable prognostic value. Moreover, METTL16 was under-expressed in PDA tissues and cell lines. Consistently, gain- and loss-of-function experiments indicated that it had a tumor suppressor role *in vitro* and *in vivo*. Further, Gene Ontology and Kyoto Encyclopedia of Genes and Genomes analyses revealed that METTL16 may have an effect on the tumor microenvironment. Notably, a markedly positive association between METTL16 expression and infiltration of B cells and CD8^+^ T cells was observed according to the CIBERSORT and TIMER databases. Enhanced expression of immune checkpoints and cytokines was elicited in patients with over-expression of METTL16. Notably, decreased expression of PD-L1 was observed when upregulation of METTL16 expression occurred in MIA PaCa-2 cells, while increased expression of PD-L1 existed when downregulation of METTL16 happened in HPAF-II cells. Collectively, these findings highlight the prognostic value of METTL16, and indicate that it is a potential immunotherapy target that could be used to regulate the tumor microenvironment and promote antitumor immunity in PDA.

## Introduction

Pancreatic ductal adenocarcinoma (PDA) is one of the most harmful neoplasms worldwide. It is the fourth most common cause of cancer deaths, and has the lowest 5-year survival rate among malignancies in the USA (Siegel et al., 2020). A similar situation occurred in 2015 in China (Chen et al., 2016). Surgical resection remains the only potentially curable treatment modality; however, this is feasible in only 10%–20% of PDA patients (Vincent et al., 2011). Chemotherapy shows little promise (de Sousa Cavalcante and Monteiro, 2014). Emerging evidence has demonstrated promising effects of immunotherapy on many malignancies (Barretina et al., 2012; Motzer et al., 2015; Sharma et al., 2016); however, it is less useful in the “cold” immune subtype of PDA (O'Reilly et al., 2019). Thus, it is imperative to explore the molecular mechanisms of tumorigenicity in PDA and identify novel therapeutic targets.

Pancreatic carcinogenesis is complex and consists of multiple processes, with important contributions from driver gene mutations. Certain important genes (KRAS, TP53, CDKN2A, and SMAD4) have been shown to be the most frequently mutated in PDA and to characterize various steps in carcinogenesis (Vincent et al., 2011). KRAS mutations, recorded as the earliest genetic disruptions, lead to chromosomal abnormalities (Buscail et al., 2020). Mutations of TP53, CDKN2A, and SMAD4 result in gene inactivation, and occur in the advanced stage of pancreatic neoplasia. However, the underlying molecular mechanism remains unclear. The basal subtype and classical subtype are two important types in PDA. Interestingly, they are characterized by the differential expression of transcription factors and downstream targets known to be important in lineage specification and differentiation during pancreatic development and regeneration. The classical subtype has high expression of adhesion-associated and epithelial genes, whereas the basic subtype shows high expression of mesenchymal genes (Collisson et al., 2011; Bailey et al., 2016).

N6-methyladenosine (m^6^A), methylation of adenosine at the N6 position, is regarded as the most prevalent and conserved internal chemical modification in eukaryotic mRNA (Desrosiers et al., 1974). Emerging evidence has indicated that m^6^A makes contributions to carcinogenesis (Wang et al., 2014; Chen et al., 2015; Geula et al., 2015; Xiang et al., 2017; Deng et al., 2018; Weng et al., 2018; Huang et al., 2020a). The deposition of m^6^A is mediated by a range of homologous factors, including methyl-transferases (also called “writers”), binding proteins (“readers”), and demethylases (“erasers”). The “writers” catalyze the formation of m^6^A. Then, the information contained in m^6^A codes can be deciphered, and a functional signal can be generated by “readers.” The methyl groups of target mRNAs can be selectively removed by “erasers.” The “writers” consist of METTL3, METTL5, METTL14, METTL16, RBM15, VIRMA, WTAP, and CBLL1. The “readers” include IGF2BP1, IGF2BP2, IGF2BP3, HNRNPC, HNRNPA2B1, YTHDC1, YTHDC2, YTHDF1, YTHDF2, YTHDF3, and RBMX. There are two “erasers,” FTO and ALKBH5 (Chen and Wong, 2020). Recent studies have found that abnormal m^6^A expression leads to tumorigenesis, such as METTL14 and ALKBH5 (Li et al., 2017b; Zhang et al., 2017; Weng et al., 2018). However, the potential role and molecular mechanism of m^6^A regulators in pancreatic tumorigenicity have not been well defined.

In this study, we explored the functional role of m^6^A regulators in the carcinogenesis of PDA. We first focused on the aberrant expression of potential m^6^A regulators under conditions when mutations of driver genes (KRAS, TP53, CDKN2A, and SMAD4) are present. METTL16 was selected uniquely for further study owing to its markedly differential expression and favorable prognostic value. *In vitro* and *in vivo* experiments indicated that METTL16 served as a tumor suppressor in PDA. Moreover, Gene Ontology (GO) and Kyoto Encyclopedia of Genes and Genomes (KEGG) analyses were conducted to explore the potential mechanism of METTL16 in PDA. We further evaluated the relationship between immune cell patterns or immune checkpoints and METTL16 expression. Collectively, these findings highlight the prognostic value of METTL16 and its potential underlying mechanism in the tumor microenvironment in PDA.

## Materials and methods

### Pancreatic ductal adenocarcinoma patients and clinical tissues

Seven pairs of PDA specimens and their counterpart nontumor clinical tissues were collected from 2019 to 2020 at Sun Yat-sen Memorial Hospital, Sun Yat-sen University (Guangzhou, China). Patients underwent no other treatment, including chemotherapy, immunotherapy, or radiological treatment before surgery. All the clinical specimens were frozen immediately and preserved at −80°C. Histological diagnosis was confirmed by two pathologists. Informed consent was obtained. This research was performed with the approval of the local ethics committee at Sun Yat-sen Memorial Hospital, Sun Yat-sen University.

### M^6^A dot blot experiment

Total RNA isolated from four PDA patients tumors specimens and adjacent nontumor tissues was extracted using RNAiso Plus (Takara, Japan, #9019) and diluted into a gradient concentration of 500 ng/μl, 250 ng/μl, and 125 ng/μl. Samples (500 ng, 250 ng, or 125 ng) that degenerated under 70°C for 2 min were deposited on an Hybond-N+ membrane (Beyotime, China, #FFN10). Then, the membrane was crosslinked by ultraviolet rays for 2 min and stained with 0.02% methylene blue (Sigma-Aldrich, USA, # M9140-25G) and washed with 75% ethanol for 30 min. The membrane was incubated with primary m^6^A antibody (1:5,000, Synaptic System, #202003) overnight at 4°C. Dot blots were visualized by autoradiography imager G: Box Chemi XT4 System (Syngene, Cambridge, United Kingdom) after incubation with secondary antibody (CST, USA, #7074S).

### Data retrieval and processing

Transcriptome data, somatic mutation data, and clinical information were collected from The Cancer Genome Atlas (TCGA) PAAD cohort (Cancer Genome Atlas Research Network. Electronic address and Cancer Genome Atlas Research Network, 2017) (https://portal.gdc.cancer.gov/). Masked somatic mutation data and expression data in FPKM format for the TCGA-PAAD cohort were obtained. Two independent Gene Expression Omnibus (GEO) datasets (GSE16515 (Pei et al., 2009) and GSE28735 (Zhang et al., 2012)) were used to detect METTL16 and ALKBH5 mRNA expression levels in PDA.

### Analysis of targeted gene expression

The expression levels of m^6^A regulators in various tumor specimens and matched normal control datasets were determined based on the ONCOMINE database (https://www.oncomine.org) (Rhodes et al., 2007). *p* < 0.05 and fold change ≥ 1.5 were used as thresholds for the ONCOMINE analysis. The “maftools” R package (version: 2.6.5) (Mayakonda et al., 2018) was used for estimation and visualization of mutation data. The expression levels of METTL16 and ALKBH5 in pancreatic cell lines were analyzed based on data from the Cancer Cell Line Encyclopedia (CCLE) (https://portals.broadinstitute.org/ccle), which includes both genomic and transcriptomic profiles for more than 1,000 cell lines (Barretina et al., 2012). The protein expression of METTL16 between healthy control and PDA tissues was compared using the HPA database (https://www.proteinatlas.org/) (Uhlen et al., 2015).

### Cell culture and transfection

Six PDA cell lines (Capan-2, MIA PaCa-2, PANC-1, SW1990, BxPC-3, and HPAF-II) and the immortalized pancreatic cell line hTERT-HPNE were purchased from the Cell Bank of the Chinese Academy of Sciences. *Mycoplasma* contamination tests were negative, and short tandem repeat assays were performed to authenticate the cell lines. MIA PaCa-2, PANC-1, and hTERT-HPNE cells were seeded in high-glucose Dulbecco’s modified Eagle medium (DMEM; Gibco, USA). HPAF-II cells were plated in minimal essential medium (Boster, USA). SW1990, Capan-2, and BxPC-3 cells were grown in RPMI-1640 (Gibco, USA). The media were supplemented with 10% fetal bovine serum (FBS, Gibco, USA), and cells were cultured at 37 °C under 5% CO_2_.

The METTL16 overexpression (OE) vector was purchased from Genechem (Shanghai, China). Short hairpin RNAs (shRNAs) against METTL16 were constructed by Genechem (Shanghai, China). For stable transfection, 2 × 10^5^ cells per well were plated in six-well plates 24 h in advance, then transfected with a 2E+6TU overexpression or shRNA plasmid using polybrene (Solarbio, China), followed by selection with puromycin (Solarbio, China). The shRNA sequences are listed in Supplementary Table S1.

### Quantitative PCR detection

Total RNA was isolated using RNAiso Plus (Takara, Japan) following the manufacturer’s instructions; then, reverse transcription was carried out using PrimeScript™ RT Master Mix (Takara, RR036A). Subsequently, qPCR was performed with TB Green™ Premix (Takara, Japan). The cycling conditions were 95°C for 30 s, 95°C for 5 s, and 60°C for 30 s for 40 cycles, with a 10 μl sample volume, using CFX Connect (Bio-Rad, USA). β-Actin was used as an endogenous control. The 2^−ΔΔCt^ method was used to evaluate expression levels (Livak and Schmittgen, 2001). The primer sequences are listed in Supplementary Table S1.

### Cell proliferation detection

The cell counting kit-8 (CCK-8; APExBIO, USA) assay was performed to estimate the cell growth capacity. A total of 2 × 10^3^ cells per well were seeded in 96-well plates in 100 µl medium. Afterward, the culture medium and CCK-8 were blended at a ratio of 10:1, and 100 µl of the resulting mixture was added to each well at different time points (24, 48, 72, and 96 h) after seeding. After incubation for 4 h, the relative absorbance was assessed at 450 nm with a Multiskan Mk3 microplate reader (Thermo Fisher, USA).

### Wound healing assay

A total of 5 × 10^5^ cells were grown with complete medium in a six-well plate. When the culture reached 90% confluency, scratches were made in the cell layer using the tip of a 10-μl sterile pipette. Afterward, cells were washed with phosphate-buffered saline, which was then replaced with serum-free culture medium, followed by incubation for another 24 h. Images of the plates were acquired using an inverted microscope, Olympus IX73 (Olympus, Japan) at two time points (0 and 24 h). The migration rate was analyzed by calculating the width by ImageJ software. It was calculated as follows: migration rate (%) = (A0–A24)/A0 × 100%, where A0 exerted as the area of the wound area at 0 h, A24 represented the remaining area of wound at 24 h.

### Transwell assays

Cell migration evaluation was estimated using transwell inserts (8-μm pore, Costar, USA). DMEM with 10% FBS (800 µl in total) was used to fill the lower insert. Cells were re-suspended, gathered, and seeded in serum-free DMEM. The DMEM (200 μl, serum-free) with cells (4 × 10^4^) was seeded into the upper insert. Cells were cultured for 24 h at 37°C before being fixed. The non-migrated cells were removed. Cells were fixed with paraformaldehyde and dyed with crystal violet. Migration potential was assessed by calculating the number of dyed cell nuclei from three stochastic fields using an imaging microscope (Nikon NI-U, Japan).

### *In vivo* experiments

Animal experiments were performed with the approval of the Institutional Animal Care and Use Committee, Sun Yat-sen University (Guangzhou, China). The female BALB/c-nu mice (4 weeks old, three per group) were acquired from the Laboratory Animal Center of Sun Yat-sen University. MIA PaCa-2 cells with stable overexpression of METTL16 expression (OE-METTL16) and its counterpart (OE-NC) MIA PaCa-2 cells were selected for animal experiments. A total of 5 × 10^6^ cells per mice were injected into the dorsal flanks. After 5 weeks, mice were sacrificed, and xenografts were collected and weighed.

### Hematoxylin and eosin staining

Paraffin-embedded tumors isolated from mice were sliced in 4-µm thickness and stained with hematoxylin for 10 min. Slides were rinsed in running water until they turned blue, and immersed in 1% acid alcohol until they turned pink. The slides were rinsed in tap water again until they turned blue and then dehydrated using an EtOH gradient. Slides were rinsed in tap water again and then counterstained with eosin solution for 1 min before being dehydrated with an EtOH gradient. Slides were placed under a coverslip with rubber and left overnight at room temperature. Specimen images were captured using an upright microscope NIS-Elements F system of Nikon NI-U (Nikon, Japan).

### Immunohistochemistry

The tumors isolated from mice were embedded in paraffin and sliced into 4 µm for further IHC staining. The slides were incubated with Ki67 antibody (diluted at 1:100) (ab92742, Abcam) overnight at 4°C, and then incubated with secondary antibodies (PV-6000, ZSGB-BIO) at room temperature for 2 h. Then, the slides were stained with the DAB kit (ZLI-9018, ZSGB-BIO), counterstained with hematoxylin and differentiated with 1% hydrochloric acid. Specimen images were captured for three photos at random using the NIS-Elements F system of an upright microscope (Nikon NI-U, Japan) (400×), and quantified the rate of positive cells using ImageJ software.

### Survival analysis

The prognostic values of METTL16 and ALKBH5 were detected using the online database Gene Expression Profiling Interactive Analysis (GEPIA2: http://gepia2.cancer-pku.cn) (Tang et al., 2019). Patients in the TCGA-PAAD cohort were stratified according to the median of METTL16 expression in GEPIA2. Log-rank test and univariate Cox proportional hazard regression were used to generate log-rank P-values and hazard ratios (HR) with 95% confidence intervals (CI), which can be generated via “survival” R package (version 3.2-10) (Liu et al., 2018) and shown by the “forestplot” R packages (version 2.0.1) (Gordon, 2017). Factors that were significant at the 0.1 level in the univariable analysis were included in the multivariable analysis.

### Functional enrichment analysis

METTL16-related differentially expressed genes (DEGs) were screened using the “limma” package (version: 3.40.2) (Ritchie et al., 2015). The patients were divided into two groups according to high and low METTL16 expression levels, separated by the median value. Limma powers differential expression analyses for RNA-sequencing and microarray studies. Limma returned empirical Bayes moderated-t p-values and adjusted P-values (Q-value) to correct for multiple-comparison testing using the Benjamini–Hochberg method to control the false discovery rate (FDR). Genes with an FDR less than 0.05 and |log2 (fold change)| > 1.0 were defined as significant DEGs. To investigate the main functional mechanisms of METTL16 and related genes, GO and KEGG analyses were both performed using the “clusterProfiler” R package (version: 3.18.0) (Yu et al., 2012). GO results with respect to biological processes, cellular components, and molecular functions, and KEGG pathways were illustrated using bubble plots.

### Analysis of Immune Cell Signatures Correlated with METTL16 Expression.

CIBERSORT (https://cibersortx.stanford.edu/) was also used to quantify the percentages of tumor-infiltrating leukocyte subsets (Newman et al., 2015). To further enhance the power of the algorithm, only samples with *p* < 0.05 were used for subsequent analysis, and 1,000 permutations were performed for estimating the immune cell populations. Tumor Immune Estimation Resource (TIMER) (http://timer.cistrome.org/), an online database (Li et al., 2017a) for analysis and visualization of tumor-infiltrating immunocyte levels, was also used to verify the correlations between METTL16 and the abundance of immune infiltrates in the TCGA-PAAD cohort.

### Statistical analysis

Data were shown as mean ± standard deviation (SD). GraphPad Prism 8 software (San Diego, CA, United States), and RStudio (RStudio Team, 2020) were used for statistical analyses. The Shapiro-Wilk test was conducted for normality of data distribution. The Student’s t-test (two-tailed) was used for the comparison of continuous variables with Gaussian distribution, and Wilcoxon rank sum test was used without Gaussian distribution; categorical variables were analyzed by the chi-squared test or Fisher’s exact test. The correlation between METTL16 and PD-L1 was analyzed by Spearman’s rank correlation test. A *p*-value less than 0.05 was considered to indicate statistical significance.

## Results

### Screening of potential m^6^A RNA methylation genes

**FIGURE 1:**
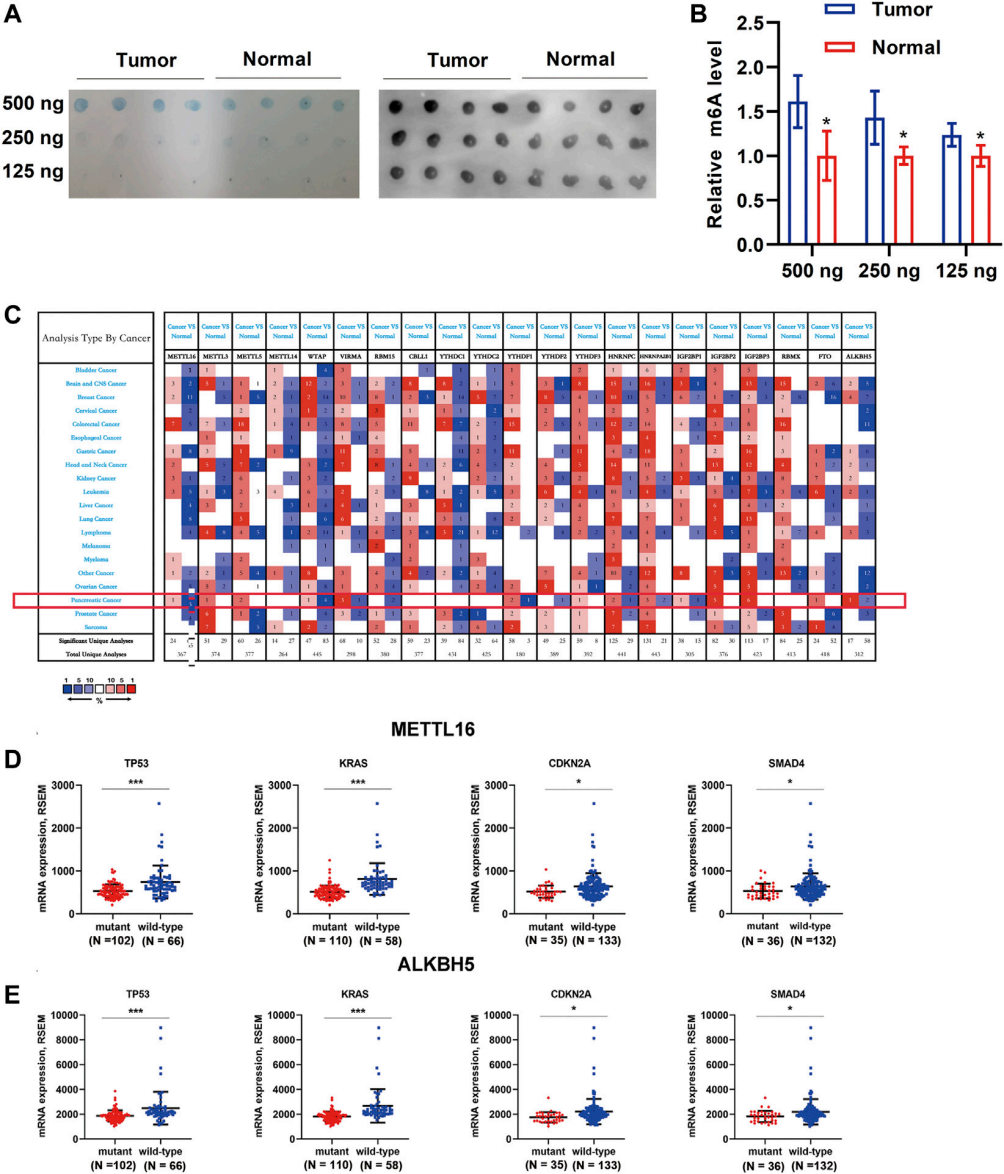
Global m^6^A levels in PDA tissue samples and transcription levels of m^6^A regulators in different cancer types. **(A)** Global m^6^A level of RNA extracted from PDA tissues (*N* = 4) and adjacent normal tissues (*N* = 4) was detected via m^6^A dot blot assays. RNAs were serially diluted and loaded equally with the amount of 500, 250, and 125 ng. Methylene blue staining (left) served as a loading control. **(B)** Histogram of m^6^A dot blot analysis indicated that an enhanced m^6^A level was observed in PDA tissues. **(C)** Analysis of transcription levels of 21 m^6^A regulators in different cancer types vs. matched normal tissues. A darker color indicates a higher number. The numbers in the colored cells represent the numbers of datasets meeting the criteria (*p*-value, 0.05; fold change, 1.5). The red pattern indicates upregulation, and the blue one indicates downregulation of genes in different analyses. Only METTL16 **(D)** and ALKBH5 **(E)** were expressed aberrantly when one of the driver genes that characterize PDA was mutated. Downregulation of METTL16 and ALKBH5 was observed in mutant tissues compared with wild-type tissues. ^*^ *p* < 0.05, ^***^ *p* < 0.001.

To determine the role of m^6^A regulators in the carcinogenesis of PDA, we first evaluated the global level of m^6^A modification in four PDA tissues and adjacent normal tissues using a dot blot assay. Interestingly, enhanced m^6^A levels were observed in PDA tissues (Figures 1A,B). Then, the ONCOMINE database was used to obtain an overall landscape of the expression of 21 RNA methylation regulators in diverse cancer types. Low expression levels of METTL16, WTAP, and ALKBH5 were observed in PDA, whereas VIRMA, METTL3, METTL5, IGF2BP2, and IGF2BP3 were highly expressed (Figure 1C). As important driver genes (KRAS, TP53, CDKN2A, and SMAD4) are frequently mutated in PDA (Supplementary Figure S1) and characterize various steps in carcinogenesis (Ryan et al., 2014; Johnson et al., 2018), we wondered whether m^6^A regulators with differential expression when these four driver genes mutation occurred would make greater contributions to pancreatic carcinogenesis. First, we identified the differentially expressed m^6^A regulators when the important driver gene (KRAS, TP53, CDKN2A, and SMAD4) mutation occurred. Only METTL16 and ALKBH5 were expressed aberrantly when the driver genes that characterize PDA were mutated. Downregulation of METTL16 and ALKBH5 was observed in mutant tissues compared with wild-type tissues (*p* < 0.05, Figures 1D,E), while other m^6^A regulators showed no differential expression when driver genes mutated (Supplementary Figures S2–S4).

### Under-expression and favorable prognostic value of METTL16 in pancreatic ductal adenocarcinoma

**FIGURE 2:**
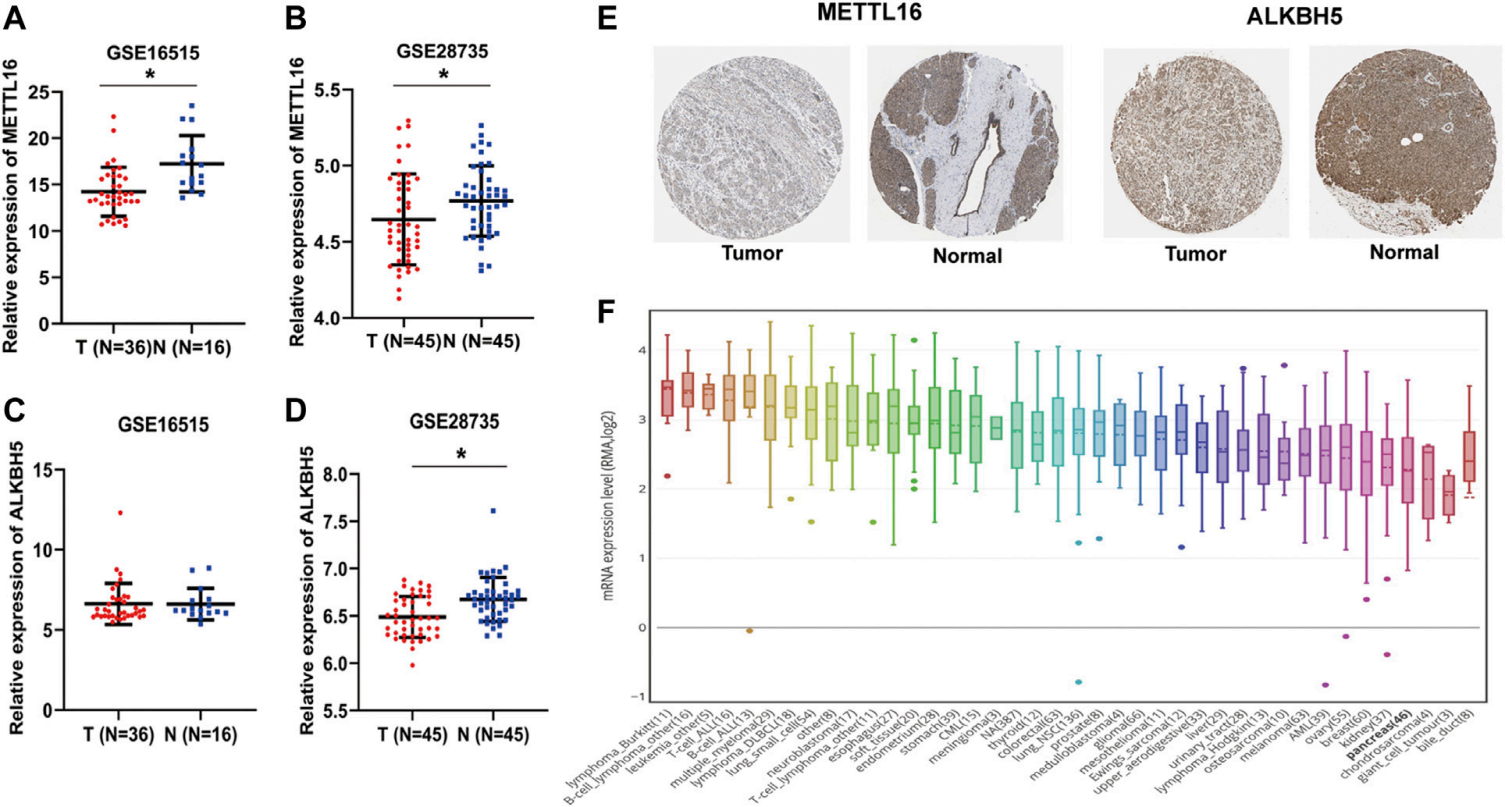
Under-expression of METTL16 and ALKBH5 in PDA. **(A,B)** Differential METTL16 expression between PDA and normal tissues in the GSE16515 **(A)** and GSE28735 **(B)** datasets (mean ± SD). **(C,D)** Differential ALKBH5 expression between PDA and normal tissues in the GSE16515 **(C)** and GSE28735 **(D)** datasets (mean ± SD). **(E)** Immunohistochemistry staining of METTL16 protein in normal pancreatic tissues and PDA in the HPA database. **(F)** Expression of METTL16 in various cancer cell lines, analyzed using the CCLE dataset.^*^ *p* < 0.05.

To further consolidate the results on the expression of METTL16 and ALKBH5 in PDA, the GEO dataset (GSE16515 and GSE28735) was used. Marked downregulation of METTL16 was observed in PDA tissues compared with adjacent nontumor tissues in both GEO datasets (*p* < 0.05, Figures 2A,B), whereas repression of ALKBH5 occurred only in GSE28735 (*p* < 0.05, Figures 2C,D). Moreover, consistent with the results for mRNA expression, protein levels of METTL16 were markedly reduced in PDA tissues according to HPA data (Figure 2E). Assessment of METTL16 and ALKBH5 in PDA cell lines was also conducted using CCLE data. Analysis of the genetic expression data from CCLE indicated that METTL16 (Figure 2F) and ALKBH5 (Supplementary Figure S5) expression levels were lower in PDA cell lines than in most other cancer cell lines.

**FIGURE 3:**
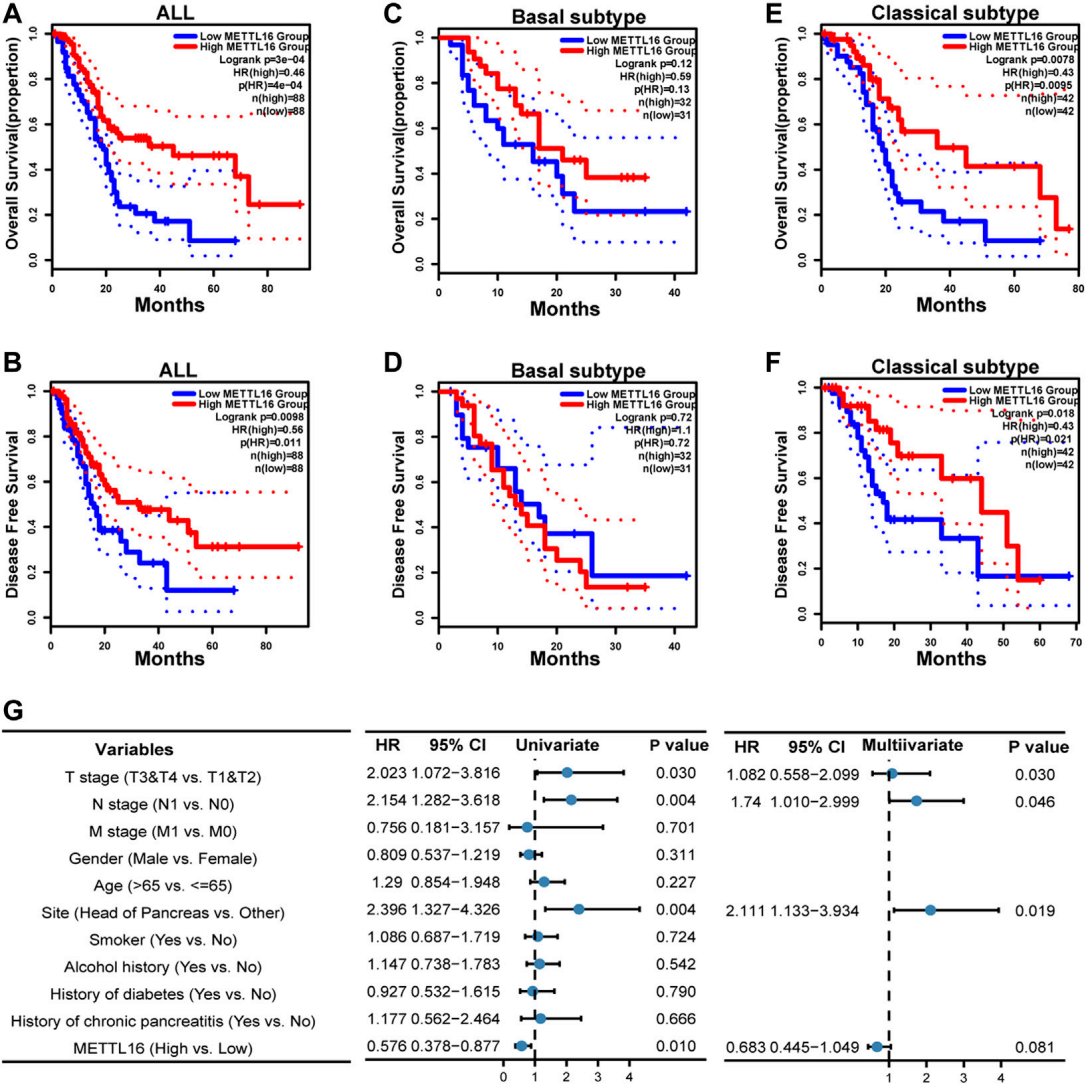
Favorable prognostic value of METTL16 in PDA. **(A,B)** Overall survival (OS) **(A)** and disease-free survival (DFS) **(B)** of all PDA patients in the TCGA cohort according to METTL16 expression. The red line represented the METTL16 high-expression group in the TCGA-PAAD cohort. The blue line showed the METTL16 low-expression group. The dotted line indicated 95% CI of the survival curves. **(C,D)** There was no significant difference in OS **(C)** or DFS **(D)** between the METTL16 high- and low-expression groups of PDA patients with basal subtype. **(E,F)** Longer OS **(E)** and DFS **(F)** were observed for PDA patients with classical subtype in the METTL16 high-expression group. **(G)** Univariate and multivariate Cox regression analyses of associations between METTL16 expression and clinicopathological characters in PDA patients.

To further evaluate the biological roles of METTL16 and ALKBH5, their prognostic value in PDA was assessed using GEPIA2. Notably, taking the median METTL16 as the cutoff value, PDA patients with higher METTL16 expression had prolonged overall survival (OS) compared with those with lower expression (*p* < 0.001, Figure 3A). Similarly, disease-free survival (DFS) was found to be significantly prolonged in the higher METTL16 expression group (*p* = 0.0098, Figure 3B). Furthermore, the correlation between METTL16 expression and survival was examined in two PDA subtypes. No significant correlation between METTL16 expression and survival was found in the basal subtype (Figures 3C,D). However, significantly longer OS (*p* = 0.0078) and DFS (*p* = 0.0018) were observed in the higher METTL16 expression group for the classical subtypes (Figures 3E,F). Meanwhile, there was no significant association between ALKBH5 expression and survival time in PDA patients (Supplementary Figure S6). Taken together, these results indicate that METTL16 is downregulated in PDA, and show a favorable prognostic biomarker in PDA patients.

To comprehend the biological function of METTL16 in more detail, the correlations between METTL16 expression and clinical information in the TCGA PAAD cohort were investigated (Supplementary Table S2). Correlations of METTL16 expression with pathologic stage (*p* = 0.001) and histologic grade (*p* = 0.014) were observed. Moreover, univariate and multivariate Cox regression analyses were conducted. These indicated that tumor site in the head of the pancreas (*p* = 0.004, HR: 2.396, 95% CI: 1.327–4.326) and severe T (*p* = 0.03, HR: 2.023, 95% CI: 1.072–3.816) and N (*p* = 0.004, HR: 2.154, 95% CI: 1.282–3.618) stage were risk factors for PDA patients, whereas upregulation of METTL16 (*p* = 0.01, HR: 0.576, 95% CI: 0.378–0.877) showed protective potential in the univariate Cox regression analysis. Consistently, tumor site in the head of the pancreas (*p* = 0.019, HR: 2.111, 95% CI: 1.133–3.934) and severe N stage (*p* = 0.046, HR: 1.74, 95% CI: 1.010–2.999) were independent prognostic factors in PDA according to the multivariate Cox regression. Although METTL16 showed no significance (HR: 0.683, 95% CI: 0.445–1.049, *p* = 0.081), a role as an independent prognostic factor could emerge if a larger sample size was used (Figure 3G).

### Tumor suppressor role of METTL16 in PDA *in vitro* and *in vivo*

**FIGURE 4:**
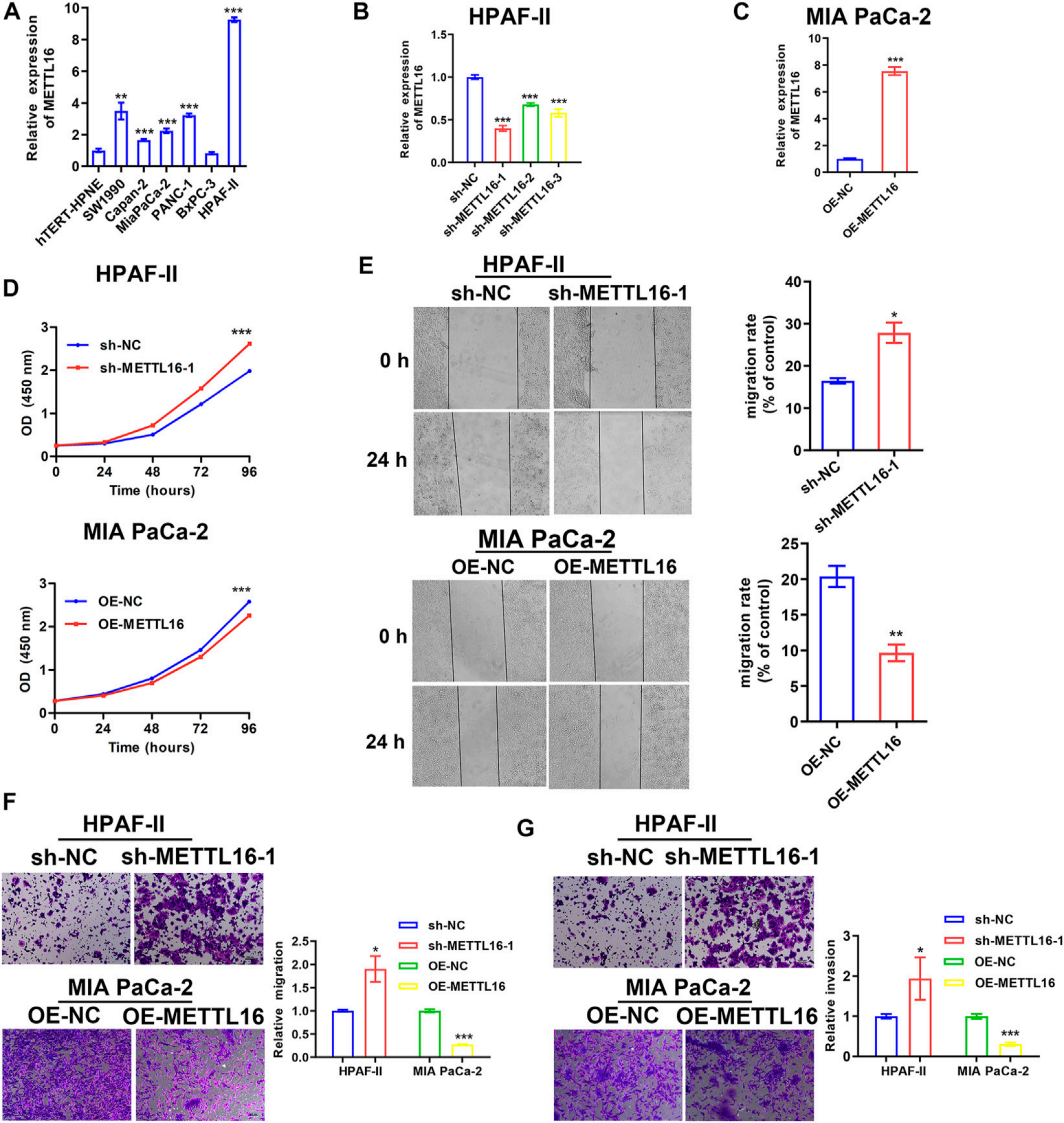
Tumor suppressor role of METTL16 in PDA *in vitro*. **(A)** Expression levels of METTL16 in PDA cell lines determined by qPCR. **(B,C)** Effective inhibition **(B)** and overexpression **(C)** systems for METTL16 were established. **(D)** Inhibition of METTL16 expression triggered cell growth (upper), whereas enhanced expression of METTL16 suppressed proliferation capacity (lower) in PDA cell lines. **(E,F)** Scratch healing assay **(E)** and transwell assay **(F)** indicated that suppression of METTL16 expression augmented migration potential, whereas upregulation of METTL16 expression attenuated migration capacity of PDA cells. **(G)** Reduction of METTL16 expression induced invasion, whereas over-expression of METTL16 inhibited invasive potential. Data are presented as mean ± SD. ^*^ *p* < 0.05, ^**^ *p* < 0.01, ^***^ *p* < 0.001.

**FIGURE 5:**
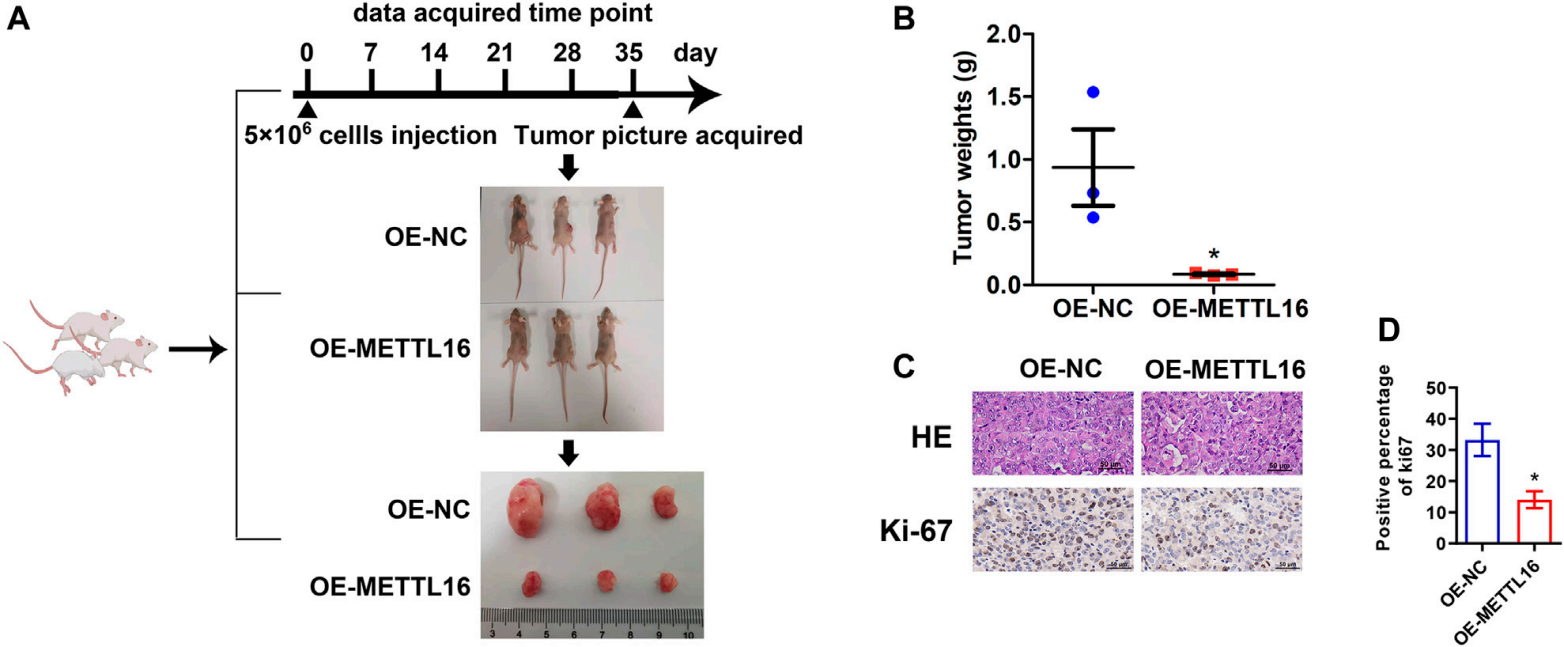
Tumor suppressor role of METTL16 in PDA *in vivo*. **(A)** Xenograft experiment revealed that enhanced METTL16 expression suppressed the proliferation capacity of MIAPaCa-2 cells. **(B)** Tumor weights for the overexpression of METTL16 (OE-METTL16) and OE-NC group (*N* = 3). **(C)** Representing immunohistochemistry staining with hematoxylin and eosin and Ki-67 staining. **(D)** Weaker Ki-67 staining was observed in the OE-METTL16 group than in the control group. Scale bar: 50 µm. Data are presented as mean ± SD. ^*^ *p* < 0.05.

As described above, METTL16 was found to be under-expressed in PDA tissues and to be a potential favorable prognostic biomarker based on analysis of various databases. We further explored the expression of METTL16 in PDA cell lines. It was shown that METTL16 expression was downregulated in most of the cell lines (SW 1990, Capan-2, BxPC-3, and MIA PaCa-2) compared with the immortalized pancreatic cell line hTERT-HPNE (Figure 4A). Accordingly, MIA PaCa-2 was selected for use in the gain-of-function assay owing to its lowest expression of METTL16, whereas HPAF-II was chosen for the loss-of-function experiment. Effective inhibition (Figure 4B) and overexpression (Figure 4C) systems for METTL16 were established. As expected, suppression of METTL16 expression augmented the growth and metastatic potential of PDA cells *in vitro*, whereas upregulation of METTL16 expression attenuated their proliferation and invasion capacity (Figures 4D–G). More importantly, overexpression of METTL16 also suppressed the growth of MIA PaCa-2 cells *in vivo* (Figure 5). These data indicate that METTL16 has a tumor suppressor role in PDA.

### Functional enrichment analysis of METTL16 in pancreatic ductal adenocarcinoma cohort

**FIGURE 6:**
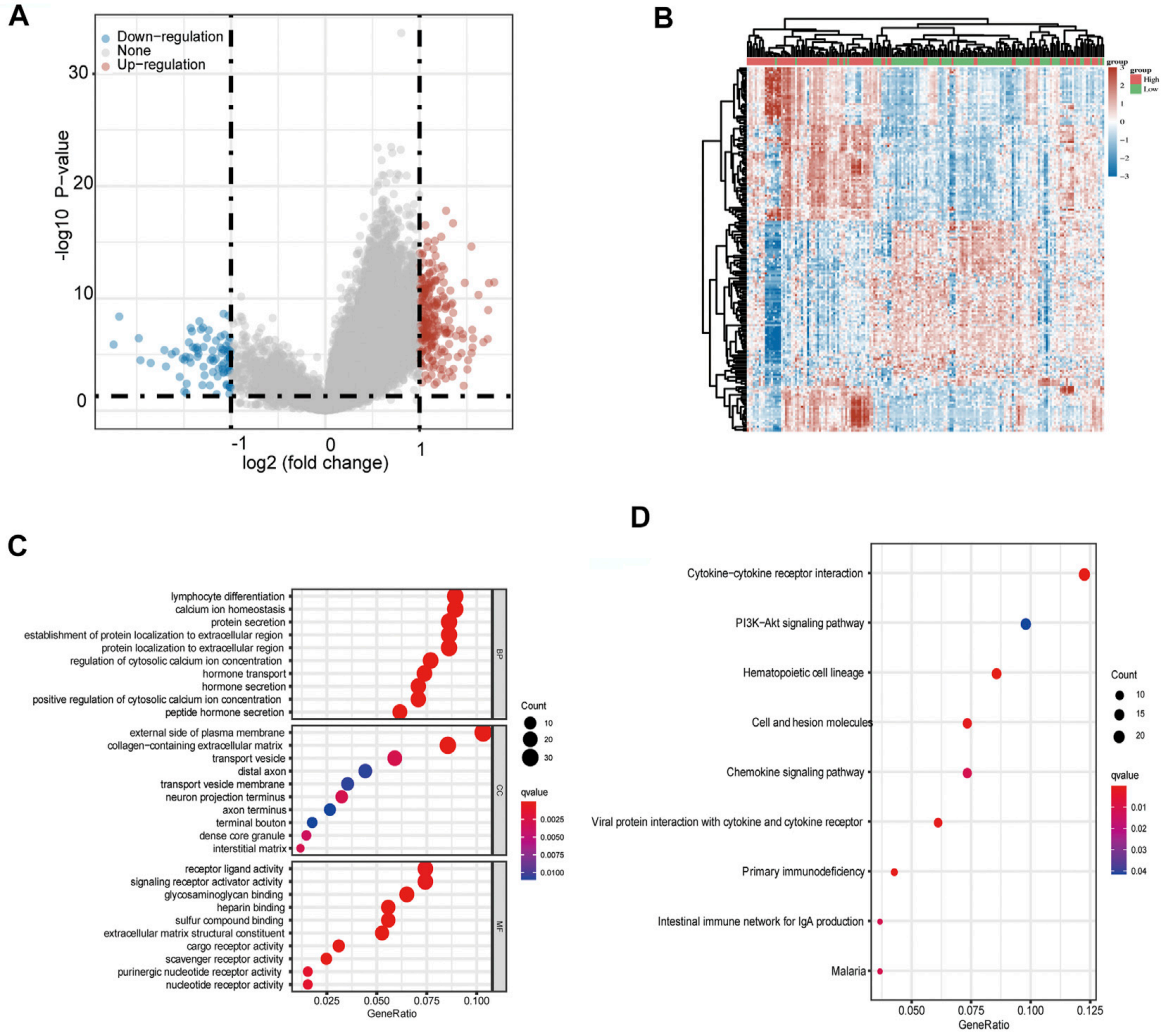
Gene enrichment analysis of METTL16 in PDA. **(A)** Volcano plots showing upregulated (red) and downregulated (blue) DEGs between METTL16-high and METTL16-low in TCGA-PAAD samples. **(B)** Heat map representation of the top 50 upregulated (red) and top 50 downregulated (blue) DEGs correlated with METTL16 in PDA. **(C)** GO analysis of DEGs between METTL16-high and METTL16-low in PDA. **(D)** Enriched KEGG pathways for DEGs between METTL16-high and METTL16-low in PDA. The X-axis represents the proportion of DEGs, and the Y-axis represents different categories. Colors indicate properties, and sizes represent the number of DEGs.

To investigate how METTL16 contributes to carcinogenesis in PDA, we conducted functional enrichment analysis in the TCGA-PAAD cohort. A total of 222 upregulated and 83 downregulated DEGs were identified with the thresholds of |log_2_ (fold change)| > 1.0 and FDR < 0.05 (Figures 6A,B). Subsequently, GO and KEGG enrichment analyses were performed. In the GO analysis, METTL16 and its DEGs were correlated with lymphocyte differentiation (biological process), the external side of the plasma membrane (cellular component), and receptor ligand activity (molecular function), with the lowest q value and highest gene expression difference (Figure 6C). The KEGG analysis showed that these genes were abundantly enriched in several pathways, including the chemokine signaling pathway, cytokine–cytokine receptor interaction, hematopoietic cell lineage, and primary immunodeficiency (Figure 6D). Collectively, these results suggest an important role of METTL16 in cancer immune response.

### Landscape of tumor-infiltrating immune cells in low- and high-METTL16 groups

**FIGURE 7:**
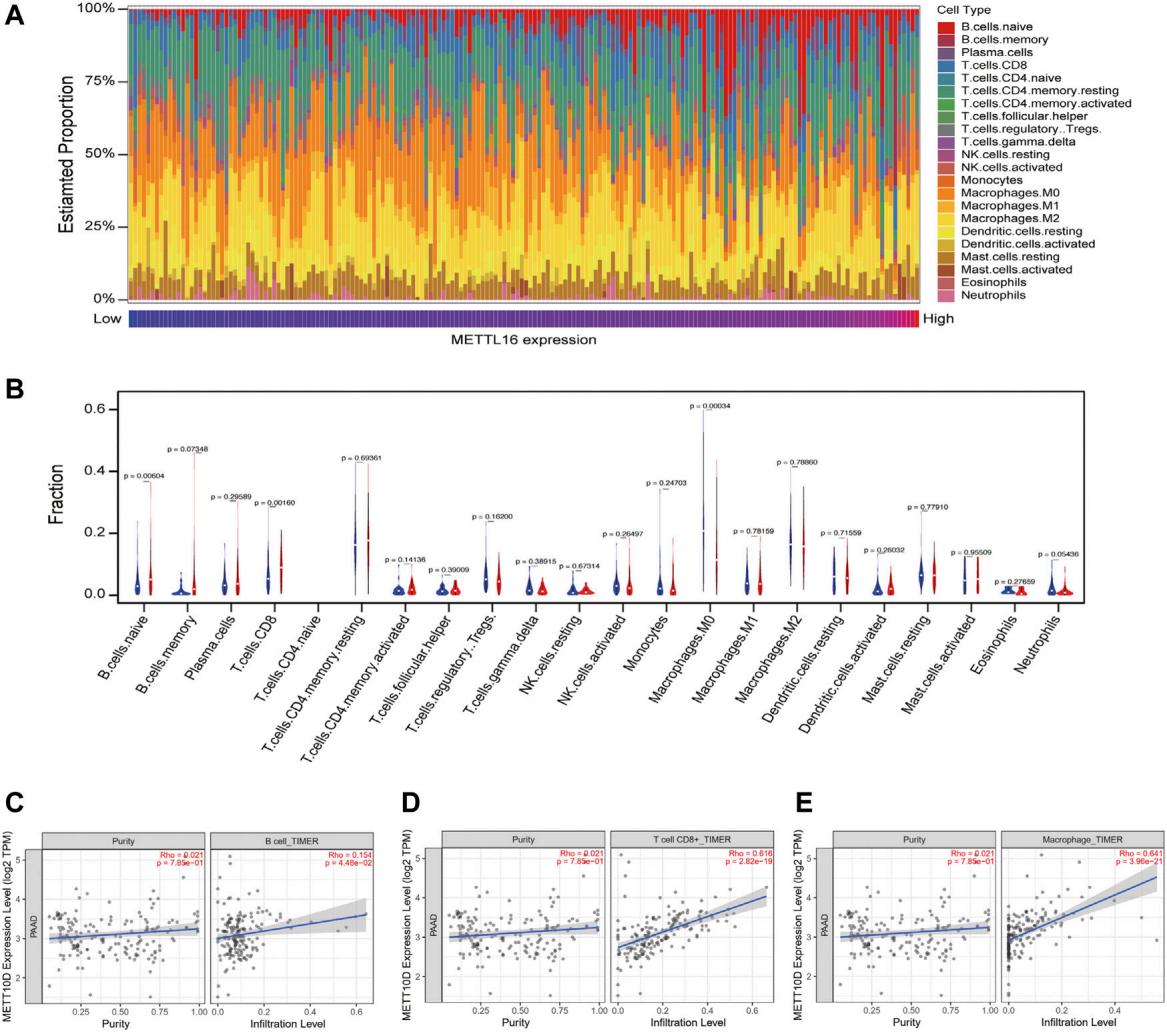
Correlation of METTL16 with tumor-infiltrating immune cells in PDA. **(A)** Immune cell infiltration profiles in the TCGA-PAAD cohort for different METTL16 expression groups. **(B)** Analysis of differential tumor-infiltrating immune cells between the METTL16 low- (blue) and high-expression groups (red) using CIBERSORT. **(C–E)** Subsequent confirmation of the relationship between METTL16 expression and tumor-infiltrating B cells **(C)**, CD8^+^ T cells **(D)**, and macrophages **(E)** via the TIMER database.

As the abovementioned results indicated that METTL16 may contribute to cancer immunity in PDA, we paid particular attention to cancer immunology when exploring its molecular mechanism. Immune cells are key players in tumor immunogenicity. Therefore, immune cell profiles were first evaluated between METTL16 high-expression and low-expression groups in the TCGA-PAAD cohort. The relative proportions of 22 immune cell components in each PDA sample were quantified using CIBERSORT (Figure 7A); these showed marked variation. Enhanced abundance of native B cells and CD8^+^ T cells was observed in the METTL16 high-expression group, whereas the abundance of M0 macrophages was reduced (*p* < 0.05, Figure 7B). Analysis using the TIMER database also showed that METTL16 expression was associated with infiltration levels of B cells, CD8^+^ T cells, and macrophages in PDA (Figures 7C–E).

### Enrichment of immune checkpoints and effector cytokines in the METTL16 high-expression group

**FIGURE 8:**
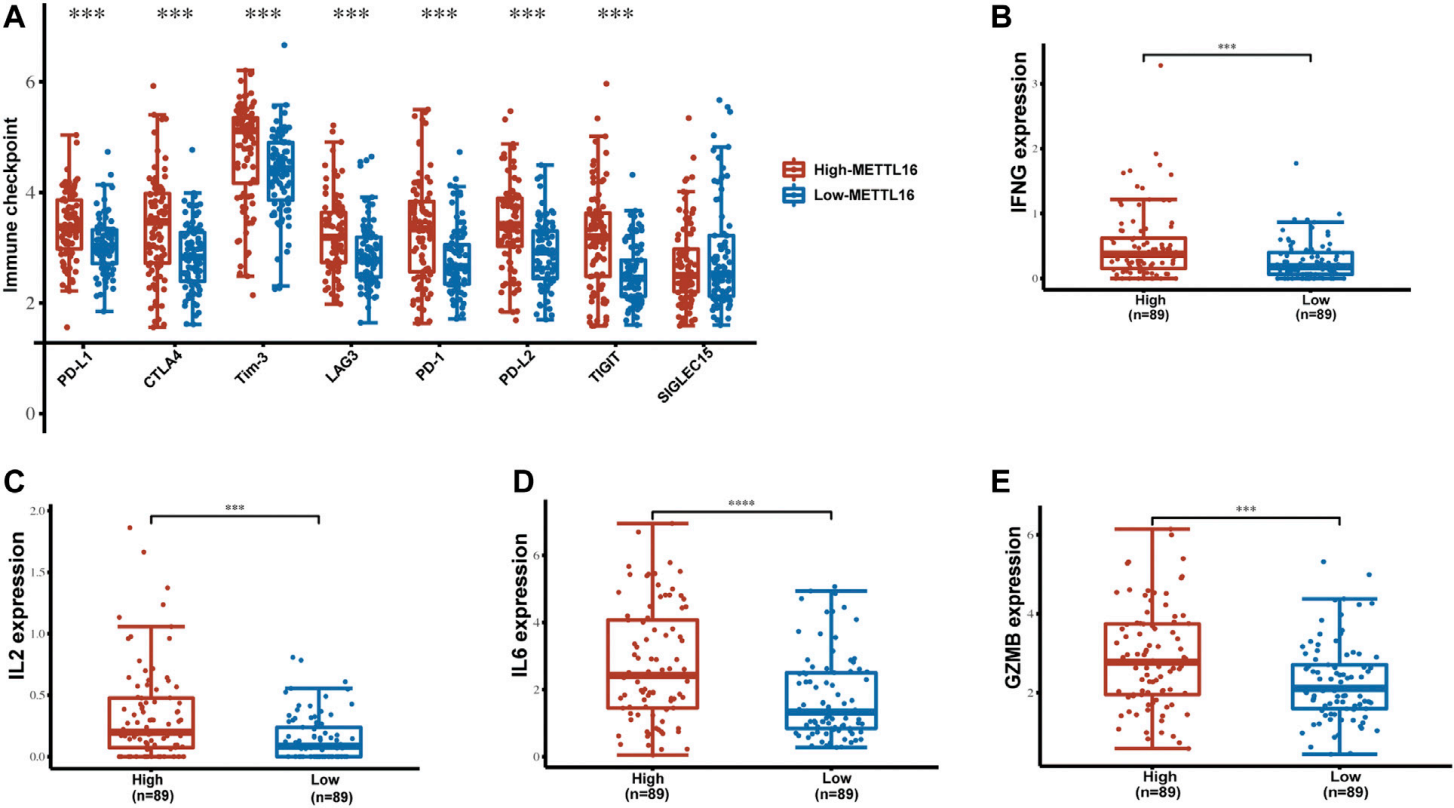
Enrichment of immune checkpoints and effector cytokines in the METTL16 high-expression group. (A) Expression distribution of immune-checkpoint–relevant transcripts in METTL16 high- and low-expression groups. **(B–E)** Analysis of effector cytokines IFN-γ **(B)**, IL-2 **(C)**, IL-6 **(D)**, and GZMB **(E)** in METTL16 high- and low-expression groups in PDA. The line within the bar marks the median, bars indicate the first and third quartiles, and whiskers above and below the box indicate the 90th and 10th percentiles. ^***^ *p* < 0.001.

**FIGURE 9:**
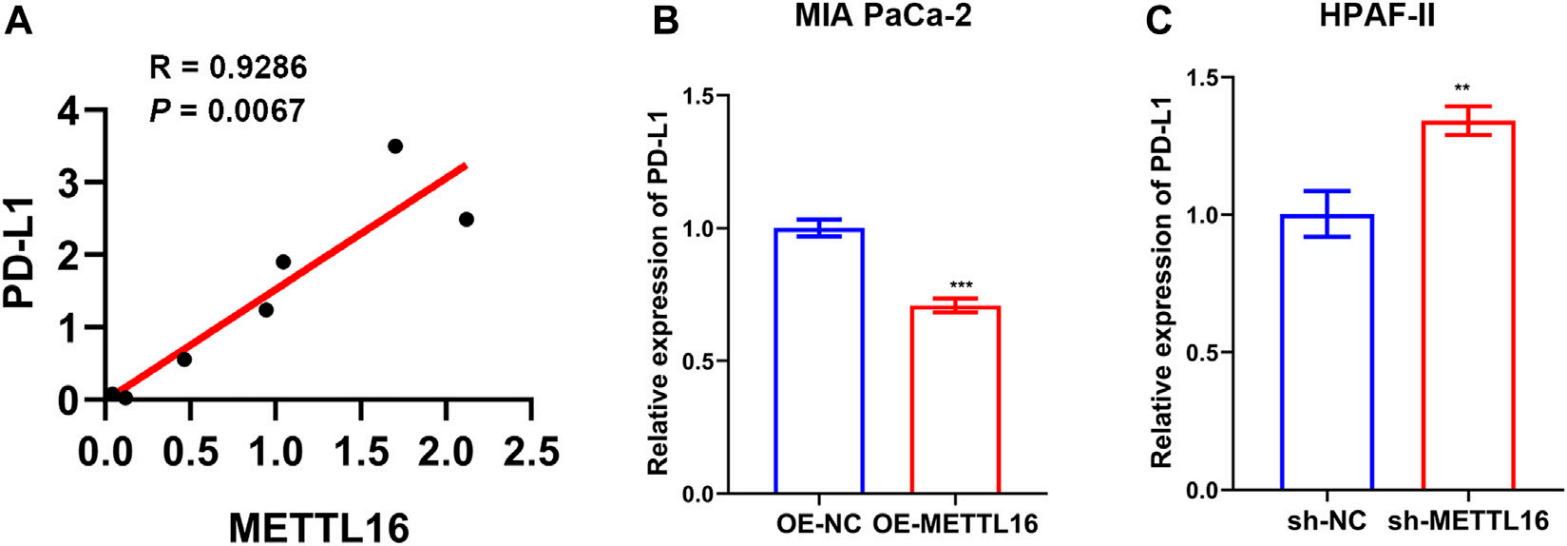
Relationship between METTL16 and PD-L1 in PDA. **(A)** A positive correlation between METTL16 and PD-L1 was observed in PDA clinical specimens by qPCR (*N* = 7). **(B)** Suppression of PD-L1 expression was observed when upregulation of METTL16 expression occurred in MIA PaCa-2 cells. **(C)** Decreased expression of METTL16 contributed to enhanced expression of PD-L1 in HPAF-II cells. Data are shown as mean ± SD. ^**^ *p* < 0.01,. ^***^ *p* < 0.001.

In addition to immune cells, immune checkpoints play a crucial part in immunotherapy. Targeting immune checkpoints has revolutionized cancer treatment strategies and effectively improved the prognosis of patients with many types of cancer. Disappointingly, however, responses to immunotherapy in PDA remain poor. To explore the relationships between METTL16 and immune checkpoint treatments, the expression levels of METTL16 and eight classical immune checkpoint genes, CTLA4, PD-1, PD-L1, PD-L2, LAG3, Tim-3, TIGIT, and SIGLEC15, were analyzed in the TCGA-PAAD cohort. Expression of seven immune checkpoint genes (CTLA4, PD-1, PD-L1, PD-L2, LAG3, Tim-3, TIGIT) showed marked elevation in the METTL16 high-expression group compared with that in the low-expression group (*p* < 0.001, Figure 8A). Cytokines are among the most crucial effectors in the immune system; therefore, the correlations between METTL16 expression levels and those of cytokines were assessed. Markedly enhanced expression of IFN-γ (IFNG), IL-2, IL-6, and granzyme B (GZMB) was observed in the METTL16 high-expression samples (*p* < 0.001, Figures 8B–E), suggesting METTL16 may prime robust cytotoxicity activity. As we know, PD-L1 is one of the most important immune checkpoint genes; thus, we wanted to further demonstrate the relationship between METTL16 and PD-L1. The qPCR experiment we performed showed that the expression of METTL16 had a positive correlation with the expression of PD-L1 in PDA clinical specimens (R = 0.9286, *p* = 0.0067, Figure 9A). Interestingly, decreased expression of PD-L1 was observed in the OE-METTL16 stable MIA PaCa-2 cell line (*p* < 0.001, Figure 9B), while increased expression of PD-L1 was detected in the sh-METTL16 stable HPAF-II cell line (*p* < 0.01, Figure 9C). Collectively, it was implied that METTL16 may play a role in regulating the expression of PD-L1 in PDA cells. In summary, these results further indicate that METTL16 may contribute to immune activity in the PDA TME.

## Discussion

Increasing evidence suggests that abnormal expression of m^6^A regulators as well as gene mutations have key roles in various malignancies, including breast cancer (Zhang et al., 2020), hepatocellular carcinoma (Zhao et al., 2018), lung cancer (Weng et al., 2018; Zhuang et al., 2020), and esophageal cancer (Zhao et al., 2021). M^6^A modification modulates almost every aspect of mRNA metabolic processes, including mRNA stability and splicing, through interactions with various reader proteins and associated complexes (Huang et al., 2020b). Dysfunction of these m^6^A regulators may cause them to function as oncogenes or tumor suppressors in malignancies including PDA. Aberrant METTL3 expression has been linked with the promotion of tumorigenesis and progression in smoking-related PDA via the Akt signaling pathway, which promotes cell proliferation, migration, and invasion (Zhang et al., 2019). METTL3-depleted cells exhibited increased sensitivity to anticancer chemoradiotherapy (Taketo et al., 2018). ALKBH5 restrained tumorigenesis in PDA by reducing methylation levels of WIF-1 and mediating Wnt signaling (Chen et al., 2017; Tang et al., 2020). YTHDF2 can accelerate the epithelial–mesenchymal transition and proliferation of PDA cells (Chen et al., 2017).

Certain important genes (KRAS, TP53, CDKN2A, and SMAD4) are most frequently mutated in PDA and characterize various steps of carcinogenesis (Li et al., 2004). Based on our results, we identified m^6^A writer METTL16 as a unique candidate that is differentially expressed among tissue samples that carry the most frequently mutated genes that characterize PDA and has potential as a favorable prognostic biomarker. METTL16 is an RNA methyltransferase that can methylate some of the adenosine residues at the N (6) position of RNAs and participates in S-adenosyl-L-methionine homeostasis by regulating the expression of MAT2A transcripts (Pendleton et al., 2017). The expression level of METTL16, together with those of other m^6^A regulators, affects the prognosis of colorectal cancer patients (Liu et al., 2019). Copy number variations of several m^6^A regulatory genes, including METTL16, are related to the OS time of patients with soft tissue sarcoma (Hou et al., 2020). Poor prognosis has been observed owing to downregulation of METTL16 in patients with hepatocellular carcinoma (Wang et al., 2020) and endocrine system tumors (Li et al., 2020). Therefore, METTL16 is considered a protective gene that can suppress the development of hepatocellular carcinoma and endocrine system tumors. However, the prognostic value of METTL16 in PDA and its potential mechanism are still unclear.

In the present study, we attempted to methodically assess the expression, prognostic value, relevance to the TME, and underlying mechanism of METTL16 in PDA. We found that METTL16 was downregulated at the transcriptional and protein levels in PDA tissues. Moreover, higher expression of METTL16 was intrinsically correlated with better prognosis in PDA patients, which may indicate that METTL16 could function as a protective factor and prognostic biomarker in PDA. GO term and KEGG pathway analyses revealed that METTL16 and associated co-expression genes mainly participated in lymphocyte differentiation, receptor ligand activity, signaling receptor activator activity, cytokine–cytokine receptor interaction, and the PI3K/Akt signaling pathway. These results suggest that METTL16 is an important player in tumorigenesis and tumor progression, with a particular role in the TME.

According to CIBERSORT analysis, the expression levels of METTL16 in PDA showed positive correlations with the abundance of naive B cells and CD8^+^ T cells and a negative correlation with that of M0 macrophages. Similarly, in our TIMER analysis, we found positive correlations between B cells, CD8^+^ T cells, and METTL16 expression. Accumulating evidence suggests that infiltration of B cells, especially naive B cells, is associated with favorable prognosis (Wang et al., 2019). Variations in T cell differentiation stage and T cell signatures would lead to different clinical outcomes. CD8^+^ T cells are believed to be the most important killer cells in antitumor immunity. They can directly kill tumor cells through specific killing of tumor cell components or by secreting IFN-γ, TNF-β, etc., to activate natural killer cells and macrophages to indirectly kill tumor cells. CD8^+^ T cells are regarded as protective against tumors, and a higher CD8^+^ T cell proportion in the PDA TME is associated with favorable outcomes (Ene-Obong et al., 2013). Previous experiments suggest that M0 macrophages accumulate in tumor tissues and can significantly increase the relative risk of death (Xu et al., 2020). These findings are consistent with our results.

In addition to adequate immune cell infiltration of the tumor, sufficient expression of immune checkpoints is required for the efficacy of immunotherapy (Hui et al., 2017). We found elevated expression of immune checkpoints, including CTLA4, PD-1, PD-L1, PD-L2, LAG3, Tim-3, and TIGIT, in the TME in the METTL16 high-expression group, which may imply that tumors with METTL16 enrichment of the TME are more likely to respond to immunotherapy with a greater level of sensitivity.

In tumor tissues, CD8^+^ T cells can be observed in unequal states of depletion, resulting in impaired antitumor cytotoxic effector function, known as T-cell exhaustion (Blank et al., 2019). Considering that the “cold” immune subtype of PDA is characterized by T-cell exhaustion, we also investigated the levels of critical effector cytokines, which can contribute to cytotoxic T lymphocyte differentiation and function. Surprisingly, we found that the classical cytokines in CD8^+^ T-cell differentiation and antitumor immunity were significantly augmented, such as IFNG, IL-2, IL-6, and GZMB (Williams and Bevan, 2007; Chen and Mellman, 2013; Johnson et al., 2018; Sledzinska et al., 2020). These findings indicate that METTL16 is a potential immunotherapy target that could be used to regulate TME and promote antitumor immunity in PDA.

## Conclusion

In summary, we found that METTL16 was downregulated in PDA tissues. Over-expression of METTL16 predicted better survival of PDA patients. *In vitro* and *in vivo* experiments indicate that it has a tumor suppressor role. Functional enrichment and dataset analysis revealed that METTL16 may participate in priming antitumor immunity in PDA.

## Data Availability

The datasets presented in this study can be found in online repositories. The names of the repository/repositories and accession number(s) can be found in the article/Supplementary Material.
