# Supplementary material for: METTL16 predicts a favorable outcome and primes antitumor immunity in pancreatic ductal adenocarcinoma
Source: Front Cell Dev Biol. 2022 Sep 9;10:759020. doi: 10.3389/fcell.2022.759020 (PMC9500295; doi:10.3389/fcell.2022.759020)
Supplement: Supplementary file 1 [file Table1.DOCX]

Supplementary Table S1 The sequences of shRNA for METTL16 and PCR Primer

| Gene | Sequence (5’-3’) | |
| --- | --- | --- |
| **shRNA** |  | |
| control | TTCTCCGAACGTGTCACGT | |
| sh-METTL16-1 | GCATAGTCGTTGTCACGACAT | |
| sh-METTL16-2 | CCAAAGTAACGTACACTGAAT | |
| sh-METTL16-3 | ATCCATGACAGTCTACAACTT | |
| **qRT-PCR** |  | |
| β-actin | F | GTGGCCGAGGACTTTGATTG |
|  | R | CCTGTAACAACGCATCTCATATT |
| METTL16 | F | AGTACCATCACCACCAAGTAAG |
|  | R | TTTCAATCCATGTCGTGACAAC |
| PD-L1 | F | GCTGCACTAATTGTCTATTGGG |
|  | R | CACAGTAATTCGCTTGTAGTCG |
