# Supplementary material for: METTL16 predicts a favorable outcome and primes antitumor immunity in pancreatic ductal adenocarcinoma
Source: Front Cell Dev Biol. 2022 Sep 9;10:759020. doi: 10.3389/fcell.2022.759020 (PMC9500295; doi:10.3389/fcell.2022.759020)
Supplement: Supplementary file 2 [file Table2.DOCX]

Supplementary Table S2. Correlation between METTL16 expression and the clinical characteristics in TCGA-PAAD patients

| Characteristic | Category | Low expression of METTL16  (n=88) | High expression of METTL16  (n=89) | p |
| --- | --- | --- | --- | --- |
| T stage, n (%) | T1 | 2 (1.1%) | 5 (2.8%) | 0.051 |
|  | T2 | 7 (4%) | 17 (9.7%) |  |
|  | T3 | 78 (44.3%) | 64 (36.4%) |  |
|  | T4 | 2 (1.1%) | 1 (0.6%) |  |
| N stage, n (%) | N0 | 21 (12.1%) | 29 (16.8%) | 0.187 |
|  | N1 | 67 (38.7%) | 56 (32.4%) |  |
| M stage, n (%) | M0 | 38 (45.2%) | 41 (48.8%) | 0.672 |
|  | M1 | 3 (3.6%) | 2 (2.4%) |  |
| AJCC Pathologic stage, n (%) | Stage I | 3 (1.7%) | 18 (10.3%) | **0.001** |
|  | Stage II | 80 (45.7%) | 66 (37.7%) |  |
|  | Stage III | 2 (1.1%) | 1 (0.6%) |  |
|  | Stage IV | 3 (1.7%) | 2 (1.1%) |  |
| Chemotherapy outcome, n (%) | PD | 23 (16.5%) | 26 (18.7%) | 0.144 |
|  | SD | 6 (4.3%) | 3 (2.2%) |  |
|  | PR | 8 (5.8%) | 2 (1.4%) |  |
|  | CR | 32 (23%) | 39 (28.1%) |  |
| Gender, n (%) | Female | 36 (20.2%) | 44 (24.7%) | 0.292 |
|  | Male | 53 (29.8%) | 45 (25.3%) |  |
| Histologic grade, n (%) | G1 | 8 (4.5%) | 23 (13.1%) | **0.014** |
|  | G2 | 53 (30.1%) | 42 (23.9%) |  |
|  | G3 | 27 (15.3%) | 21 (11.9%) |  |
|  | G4 | 1 (0.6%) | 1 (0.6%) |  |
| Age, n (%) | <=65 | 41 (23%) | 52 (29.2%) | 0.133 |
|  | >65 | 48 (27%) | 37 (20.8%) |  |

Note：Fisher exact test: T stage, M stage, AJCC Pathologic stage, Chemotherapy outcome, Histologic grade；

Chi-square test: N stage, Gender, Age；
