# Supplementary material for: METTL16 predicts a favorable outcome and primes antitumor immunity in pancreatic ductal adenocarcinoma
Source: Front Cell Dev Biol. 2022 Sep 9;10:759020. doi: 10.3389/fcell.2022.759020 (PMC9500295; doi:10.3389/fcell.2022.759020)
Supplement: Supplementary file 3 [file Image1.pdf]

Supplementary Material

Supplementary Figures

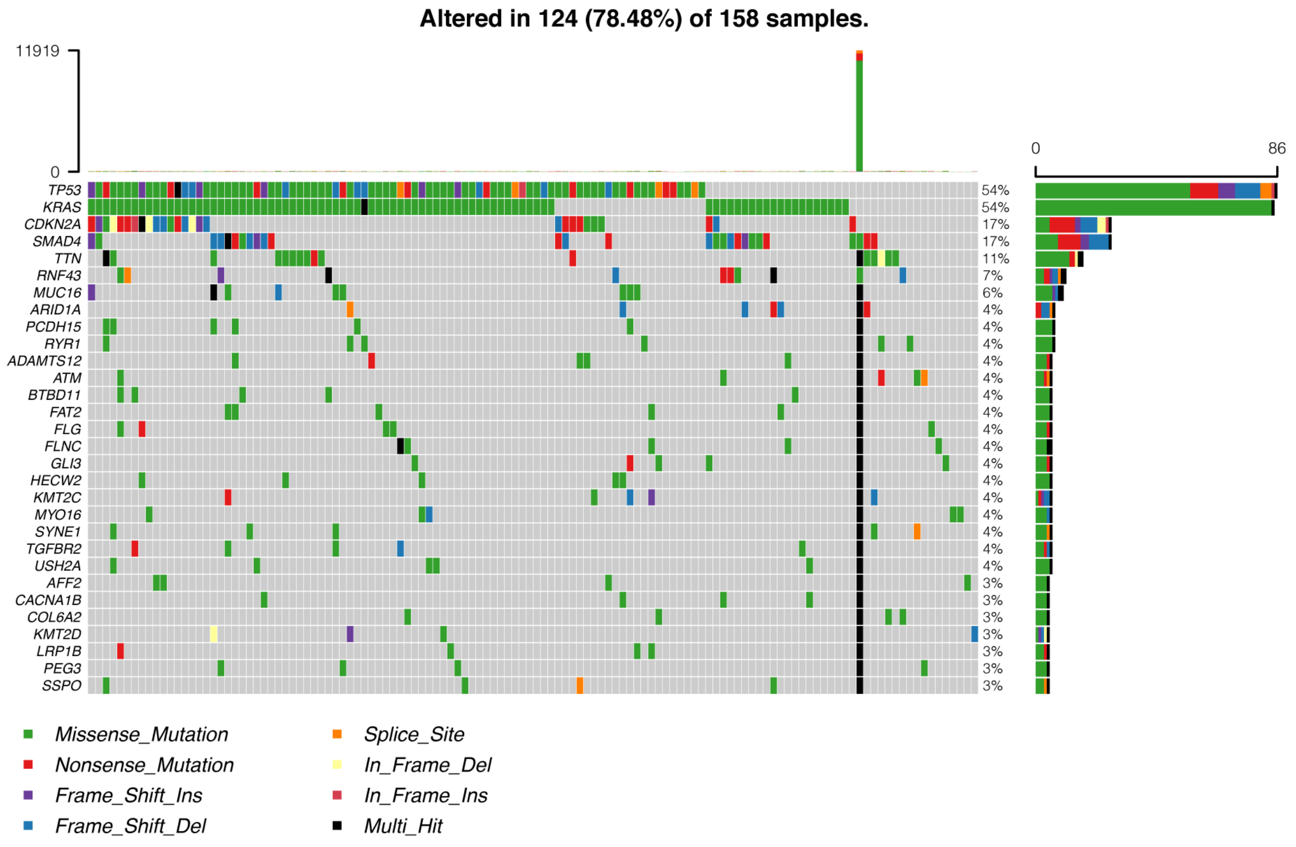

Supplementary Figure 1. Landscape of mutation profiles in PDA samples. Mutation information of each gene in each sample was shown in the waterfall plot, where different colors with specific annotations at the bottom meant the various mutation types.

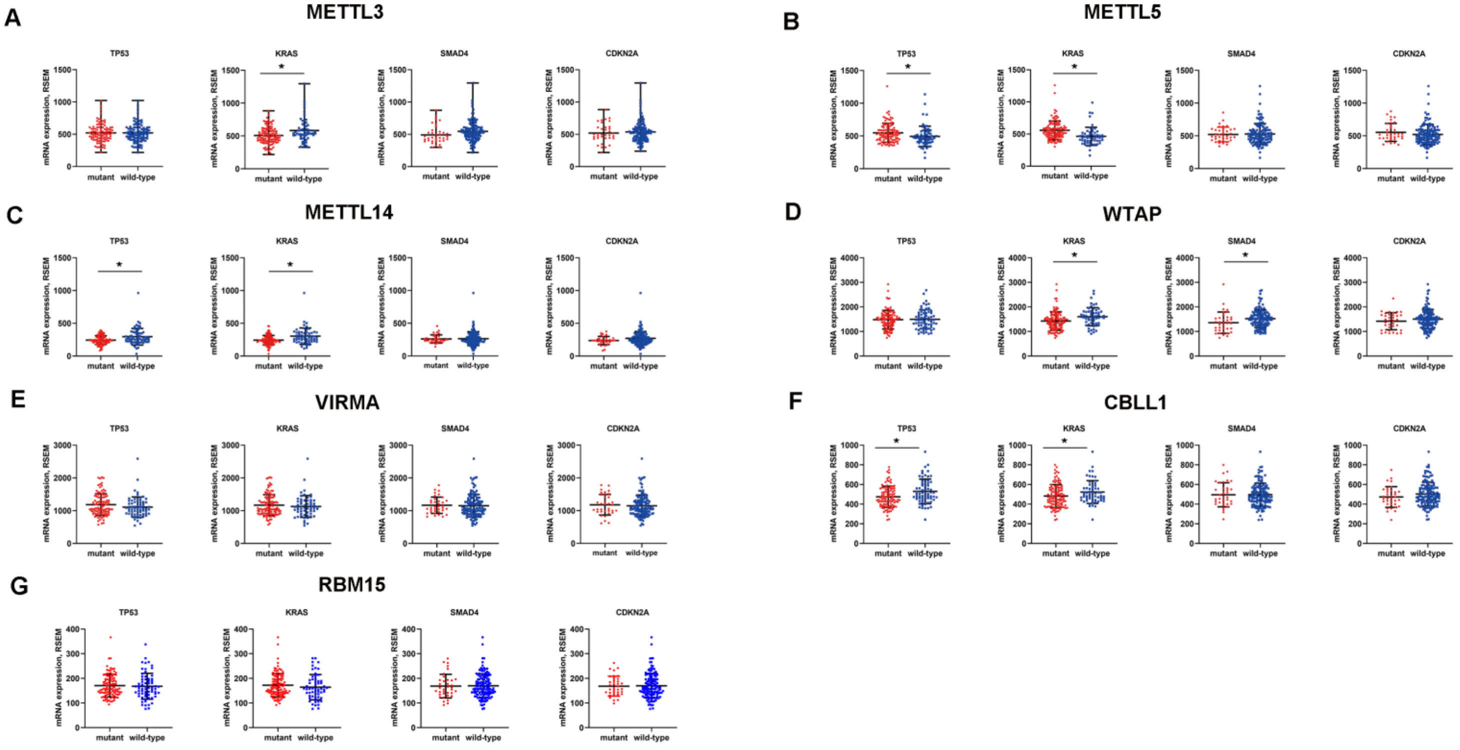

Supplementary Figure 2. The expression of m6A "writers" in TCGA-PAAD cohort between samples with a wild-type and mutated genes that characterize PDA. The expression of METTL3 **(A)**, METTL5 **(B)**, METTL14 **(C)**, WTAP **(D)**, VIRMA **(E)**, CBLL1 **(F)** and RBM15 **(G)** (mean ± SD). $^{*}P < 0.05$.

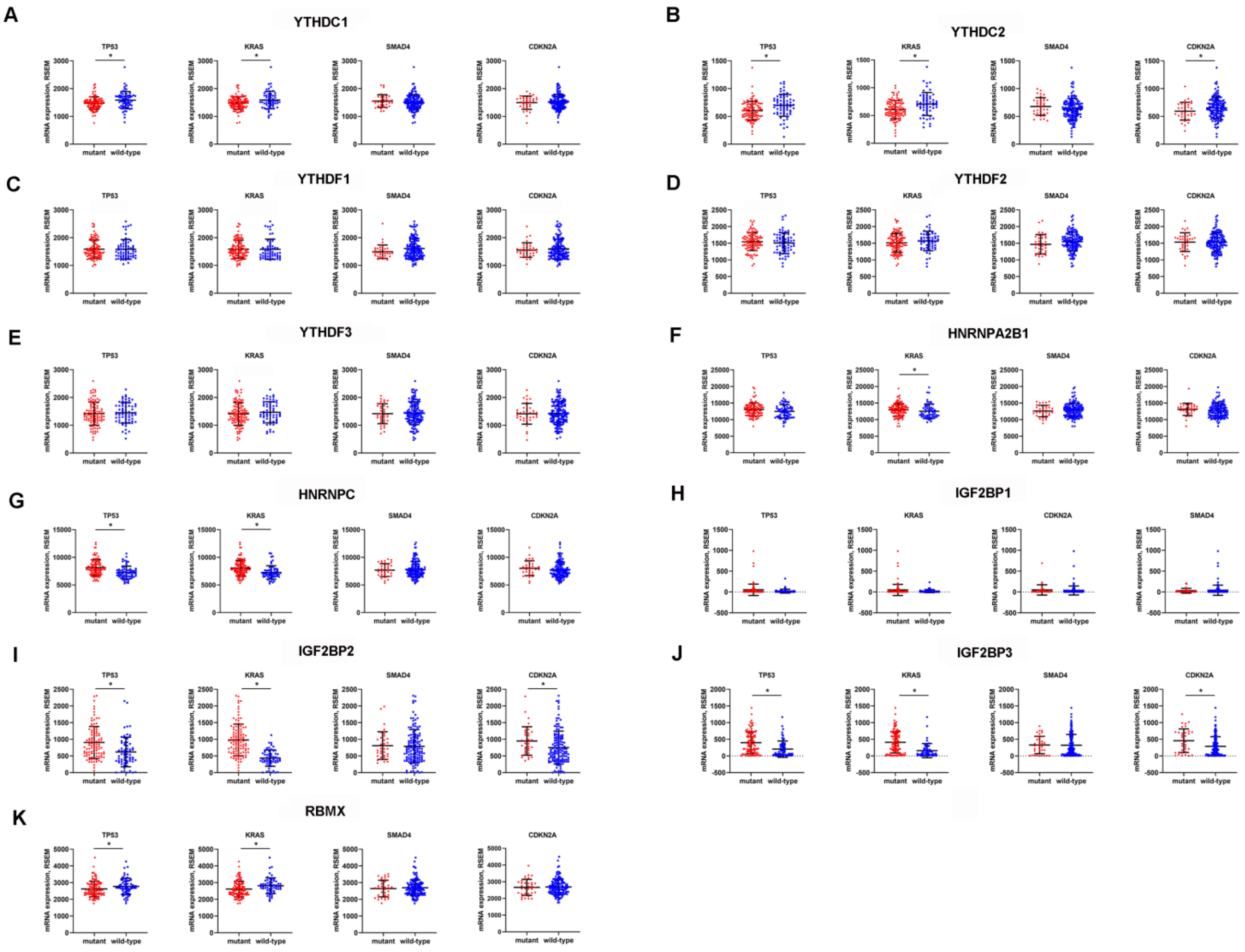

Supplementary Figure 3. The expression of m6A “readers” in TCGA-PAAD cohort between samples with a wild-type and mutated genes that characterize PDA. The expression of YTHDC1 **(A)**, YTHDC2 **(B)**, YTHDF1 **(C)**, YTHDF2 **(D)**, YTHDF3 **(E)**, HNRNPA2B1 **(F)**, HNRNPC **(G)**, IGFBP1 **(H)**, IGFBP2 **(I)**, IGFBP3 **(J)** and RBMX **(K)** (mean ± SD). $^{*}P < 0.05$.

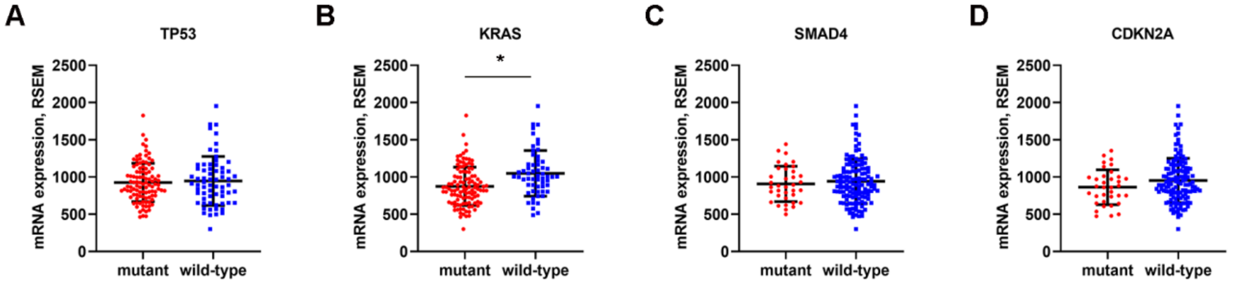

Supplementary Figure 4. The expression of m6A "eraser" FTO in TCGA-PAAD cohort between samples with a wild-type and mutated genes that characterize PDA. Data were presented as mean ± SD. $^{*}P < 0.05$.

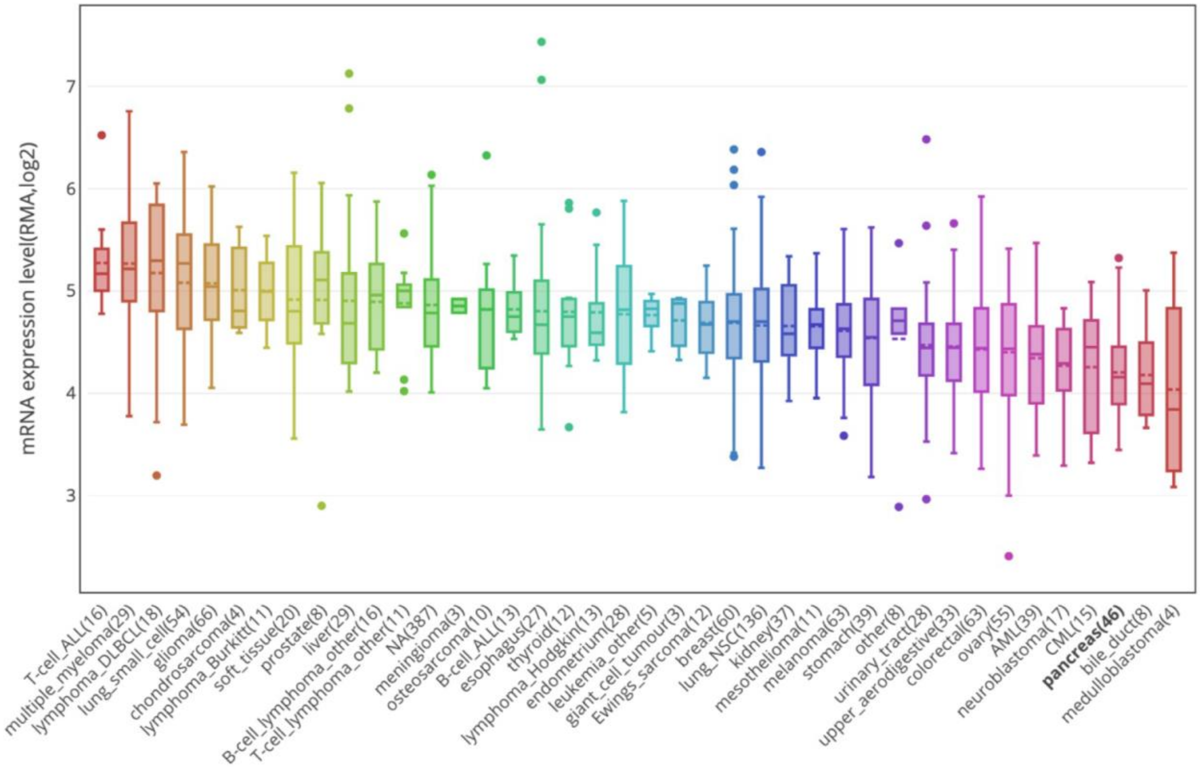

Supplementary Figure 5**.** The expression of ALKBH5 in PDA cell lines, analyzed by CCLE.

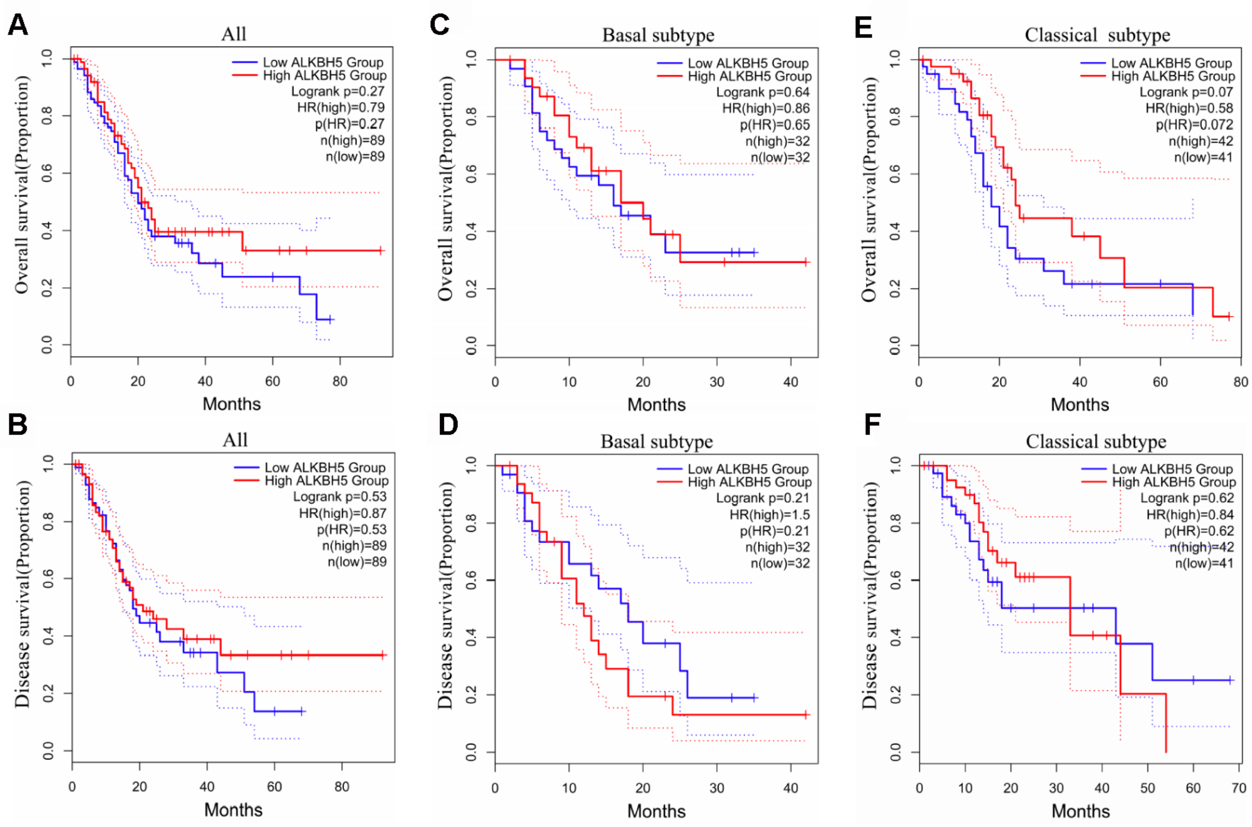

Supplementary Figure 6**. The relationship between ALKBH5 expression and prognosis in PDA.** **(A)** Overall survival (OS) and **(B)** disease-free survival (DFS) of all PDA patients in TCGA cohort based on ALKBH5 expression. **(C)** OS and **(D)** DFS in PDA patients with basal subtype based on ALKBH5 expression. **(E)** OS and **(F)** DFS in PDA patients with classical subtype based on ALKBH5 expression.
